# Supplementary figures and images for: The taxonomy of a new parvicursorine alvarezsauroid specimen IVPP V20341 (Dinosauria: Theropoda) from the Upper Cretaceous Wulansuhai Formation of Bayan Mandahu, Inner Mongolia, China
Source: PeerJ. 2015 Jun 9;3:e986. doi: 10.7717/peerj.986 (PMC4465946; doi:10.7717/peerj.986)

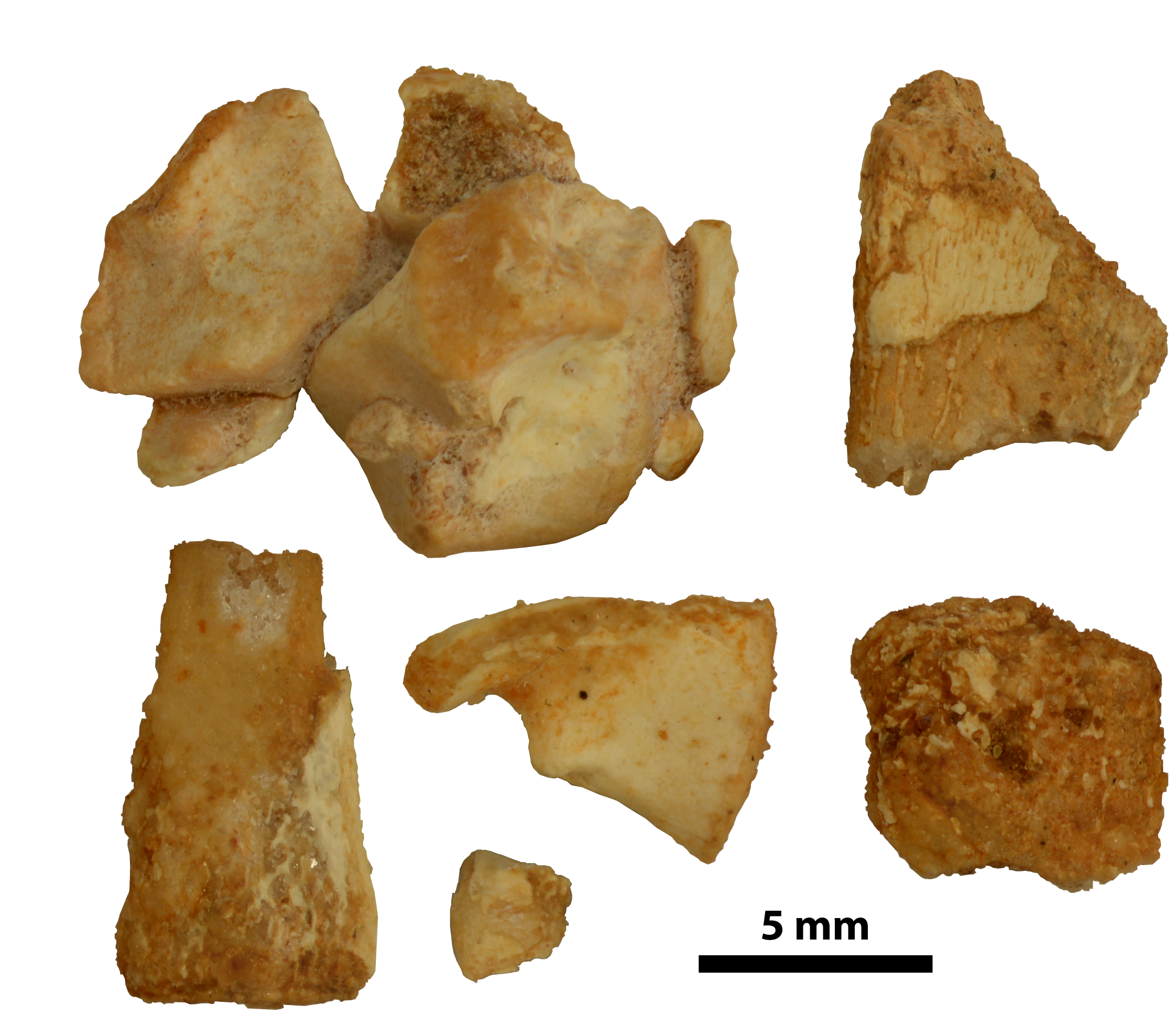

Supplement: Figure S1 — Unidentifiable bone fragments from the IVPP V20341 locality, including a probably partial centrum and potential tarsal (mammal?) or carpometacarpal bones. [file peerj-03-986-s003.png]
